# Comparison of percutaneous nephrolithotomy and flexible ureterorenoscopy in the treatment of single upper ureteral calculi measuring 1 to 2 centimeters: a retrospective study

**DOI:** 10.1186/s12894-024-01408-9

**Published:** 2024-01-28

**Authors:** Qinghua He, Xuedong Wei, Eran Wu, Raoshan Luo, Lizhi Yu, Weiming Liang

**Affiliations:** The First Affiliated Hospital of Guangxi University of Science and Technology, Guangxi University of Science and Technology, 124 Yuejin Road, Liuzhou, 545000 Guangxi Province China

**Keywords:** Micropercutaneous nephrolithotomy, Flexible ureteroscopy, Complication, Hospitalization time, Operation time, Stone free rate

## Abstract

**Purpose:**

To compare the efficacy and safety of micropercutaneous nephrolithotomy (MPCNL) and flexible ureteroscopy (FURS) in the treatment of single upper ureteral calculi measuring 1 to 2 centimeters.

**Methods:**

This study is a retrospective analysis that combines a review of medical records with an outcomes management database. A total of 163 patients who underwent MPCNL and 137 patients who had FURS were identified between January 2017 and December 2021. Demographic data, operation time, hospitalization time, stone-free rate, and complication rate were collected and analyzed.

**Results:**

Preoperative general data of sex, age, BMI, serum creatinine, time of stone existence, stone hardness, stone diameter, preoperative hydronephrosis, and preoperative infection of the MPCNL group have no statistically significant difference with that of the FURS group. All MPCNL or FURS operations in both groups were successfully completed without any instances of reoperation or conversion to another surgical procedure. Patients who underwent MPCNL had a considerably reduced operation time (49.6 vs. 72.4 min; *P*<0.001), but a higher duration of hospitalization (9.1 vs. 3.9 days; *P*<0.001) compared to those who underwent FURS. The stone-free rate in the MPCNL group was superior to that of the FURS group, with a percentage of 90.8% compared to 71.5% (*P*<0.001). There was no statistically significant disparity in the rate of complications between the two groups (13.5% vs. 15.3%; *P* = 0.741).

**Conclusion:**

Both MPCNL and FURS are viable and secure surgical choices for individuals with solitary upper ureteral calculi measuring 1 to 2 cm. The FURS procedure resulted in a shorter duration of hospitalization compared to MPCNL. However, it had a comparatively lower rate of successfully removing the stones and required a longer duration for the operation.There were no substantial disparities observed in the complication rate between the two groups.FURS is the preferable option for treating uncomplicated upper ureteral calculi, whereas MPCNL is the preferable option for treating complicated upper ureteral calculi.Prior to making treatment options, it is crucial to take into account the expertise of surgeons, the quality of the equipment, and the preferences of the patient.

**Trial registration:**

No.

## Introduction

Urinary lithiasis, a prevalent condition of the urinary system, can lead to disruption of renal function and potentially life-threatening sepsis, particularly when higher ureteric stones are present [1, 2]. Extracorporeal shock wave lithotripsy (SWL), ureterorenoscopy (URS), percutaneous nephrolithotomy (PCNL), and ureterolithotomy are frequently used as procedures. However, there are variations in the rates of stone clearance and complications associated with each procedure [3, 4]. The best treatment of upper ureteral calculi has long been controversial, but the ultimate aim is to achieve complete stone removal with minimal morbidity [5].

SWL was the chosen treatment procedure for patients with upper ureteral calculi smaller than 10 mm. If SWL was not successful or if the stone was larger than 10 mm, URS or PCNL were utilized as alternate therapy options [6]. Ureterolithotomy is considered as the final option when alternative treatments have proven ineffective or unsuccessful [5].

Micropercutaneous nephrolithotomy (MPCNL) is described as modified PCNL in which percutaneous single-step percutaneous renal access and stone fragmentation are performed under direct vision through a specialized optical puncture system called an “all-seeing needle” through a 4.8-Fr sheath [7, 8]. Due to its reduced dimensions and the ability to execute in a single step without the need for tract dilatation, MPCNL offers a smaller tract size and lower related morbidity compared to PCNL. Furthermore, with the recent advancements in retrograde flexible ureteroscopy (FURS) and laser technology, it has become more feasible to treat upper ureteral calculi by utilizing the natural lumen [9].

The objective of this study was to retrospectively assess the effectiveness and safety of MPCNL and FURS in treating upper ureteral calculi measuring 1 to 2 centimeters.

## Methods

### Study design

This study is a retrospective analysis that combines a review of medical records with an outcomes management database. Data for this database was gathered on all patients who had a single upper ureteral calculi and met the specified criteria for inclusion between January 2017 and December 2021.

### Ethical approval and consent

The study was carried out in accordance with the Declaration of Helsinki and the International Conference on Harmonisation Tripartite Guideline on Good Clinical Practice. Prior to participation, all patients furnished signed informed consent. The Ethics Committee of the First Affiliated Hospital of Guangxi University of Science and Technology granted approvals in December 2021 (approval number: 2021-LC053).

### Patients

Inclusion criteria: calculi larger than 10 mm and shorter than 20 mm in diameter,located in single upper level of upper ureter(from ureteropelvic junction to parapophysis of fourth lumbar vertebra) ,underwent MPCNL or FURS,age ≥ 18 years. Exclusion criteria: patients with a previous ipsilateral history of renal ureteral surgery; patients requiring simultaneous treatment of renal stones or bilateral ureteral stones; patients with severe underlying diseases, coagulation abnormalities or isolated renal renal malformations; patients with a solitary kidney, ureteropelvic junction obstruction, pelvic kidney abnormalities; patients with non-opaque and multiple stones. A total of 163 patients who received MPCNL and 137 patients who underwent FURS were discovered from January 2017 to December 2021 using our hospital’s electronic database.

### Procedure of MPCNL and FURS

Prior to surgery, all patients underwent preoperative urinary CT, intravenous urography, urine routine, urine culture, blood routine, coagulation function, and creatinine level assessments. All patients received preoperative prophylactic antibiotics. Patients who had positive urinary bacterial cultures were administered appropriate antibiotics, and the surgical procedure was conducted once the infection was well managed.

Both FURS and MPCNL are the surgical options available for patients. Typically, we suggest utilizing FURS as the primary method for managing straightforward upper ureteral calculi, while MPCNL is the ideal choice for addressing complex upper ureteral calculi. Nevertheless, the cost played a crucial role in shaping patients’ choices regarding their treatment. The cost of the MPCNL surgery is approximately 12,000 RMB, whereas the FURS procedure is around 27,000 RMB. The patients ultimately made the final decision.

During the MPCNL surgery, the patient was positioned in lithotomy after being given general anesthesia. A 5 F ureteral catheter was then introduced into the urethra and guided by fluoroscopy into the renal pelvis. The patient’s collecting system became enlarged and filled with a contrast agent to simulate artificial hydronephrosis after the patient was repositioned to a semi-oblique supine position at an angle of 30°~45°. A puncture is performed at the middle calyx, either subcostally or supracostally, with the use of ultrasound imaging. A trilateral connector was affixed to the proximal end of the sheath to facilitate the insertion of the telescope and the connection of the laser fiber and irrigation system, after the removal of the inner needle with stylet. The stone was fully disintegrated using a holmium laser. Fragment removal was achieved through either flushing or forceps extraction. At the conclusion of the procedures, a nephrostomy tube was inserted beside a 6 F ureteral stent. Typically, the tube was securely fastened and taken out on the third day following the operation, and the stent was removed 2–4 weeks later by the use of cystoscopy.

In the FURS procedure, a 6 F Double J(DJ) ureteral stent is inserted two weeks before the procedure to passively dilate the ureter. This is done in complicated situations with an impacted stone or a restricted ureteral orifice. Following the administration of general anesthesia, the patient is positioned in a dorsal lithotomy and a mild Trendelenburg position. The F7 rigid ureteroscope was used to see the bladder clearly, and then the DJ ureteral stents were taken out. A hydrophilic guide wire is used to install a 12 F ureteral access sheath under the F7 hard ureteroscope. The ureteral access sheath facilitates convenient and repeated access to the upper urinary tract. In addition, the utilization of a ureteral access sheath can reduce intrarenal pressure and enhance visual clarity by providing a consistent outflow. A gentle ureteroscope was inserted, and the ureter was meticulously examined to determine the precise position of the stones. The stone was thoroughly disintegrated using a holmium laser. Fragment removal was achieved through either flushing or forceps extraction. A 6 French stent is inserted and subsequently extracted during a period of 2–4 weeks post-surgery by the utilization of cystoscopy.

The same surgical team performed both procedures. The operating surgeon conducted postoperative follow-up, with the first appointment occurring between weeks 2–4 and the second appointment at 3 months after the surgery.

### Data collected

Demographic data, such as name, date of birth, BMI, and gender, were obtained from the electronic database of our hospital. The study involved examining the medical records of hospitalized patients, specifically their admission notes, progress notes, operation dictations, and discharge summaries. The purpose was to gather information on the date of admission, date and time of surgery, date of discharge, duration of stone presence, type of surgery performed, and any complications that occurred. The preoperative urinary CT was used to assess the stone hardness, stone diameter, and preoperative hydronephrosis. The absence of stones was ascertained with a urinary CT scan conducted three months after the surgery. The surgery was deemed effective if there were no remaining stones. The complications were categorized according to the Clavien-Dindo grading system [10]. Sepsis refers to an atypical systemic reaction to an infection that is often ordinary. It involves an exaggerated inflammatory response, followed by a phase of weakened immune response and failure of several organs.

### Statistical analysis

The Statistical Package for the Social Science (SPSS,version 24.0) was used for statistical analysis of the data. Numerical variables are expressed as mean ± standard deviation. The significance of differences between the two groups was tested with the Mann-Whitney U test. Nominal variables were tested with Pearson’s c^2^ test. Differences were considered significant at *P* < 0.05.

## Results

Characteristics of the two groups at baseline are given in Table 1. In the two groups, the comparative differences in preoperative general data of sex, age, BMI, serum creatinine, time of stone existence, stone hardness, stone diameter, preoperative hydronephrosis and preoperative infection were not statistically significant (*P* > 0.05) .

**Table 1:** Characteristics of Patients at Baseline

parameters	MPCNL(*n* = 163)	FURS(*n* = 137)	*P* value
Age (mean ± SD, year)	53.9 ± 14.3	51.4 ± 12.4	0.220
Sex (M/F)	98/65	80/57	0.814
BMI(mean ± SD, kg/m2)	24.54 ± 3.61	24.39 ± 3.64	0.674
Serum creatinine (mean ± SD, umol/L)	77.9 ± 28.1	78.5 ± 25.9	0.803
Side(right/left)	76/87	79/58	0.261
Stone hardness(mean ± SD, HU)	892.9 ± 223.3	910.1 ± 197.2	0.474
Stone diameter(mean ± SD ,mm)	13.21 ± 1.88	13.16 ± 1.77	0.834
Stone impaction	48	32	0.201
Preoperative hydronephrosis	57	37	0.196
Preoperative infection	50	32	0.193

All of the MPCNL or FURS surgeries in two groups were successfully completed, with no one returned to opening or other surgery.The data in Table 2 shows the differences with respect to outcomes between the two groups. Patients treated by MPCNL had significantly shorter operation time (49.6 vs. 72.4 min; *P*<0.001) but longer hospitalization time (9.1 vs. 3.9 days; *P*<0.001) than those treated by FURS.Stone free rate in the MPCNL group was better than that of the FURS group(90.8% vs. 71.5%; *P*<0.001).

**Table 2:** Outcomes in the MPCNL and FURS

Parameters	MPCNL(*n* = 163)	FURS(*n* = 137)	*P* value
Operation time(mean ± SD, min)	49.6 ± 10.7	72.4 ± 14.4	<0.001
Hospitalization time (mean ± SD, day)	9.1 ± 2.2	3.9 ± 1.2	<0.001
Stone free rate	90.8%(148/163)	71.5%(98/137)	<0.001
Complication rate	13.5%(22/163)	15.3%(21/137)	0.741

There was no statistically significant difference in complication rate between two groups(13.5% vs. 15.3%;*P*=0.741). Based on the Clavien-Dindo grading system,no complication over grade IV was encountered in the present study.A total of 22 complications were observed in the MPCNL group, including 3 cases of renal pelvic injury(grade I), 5 cases of renal colic(grade I), 4 cases of fever over 38.5 ℃(grade II), 9 cases of hemorrhage(grade II) who was controlled with blood transfusion, 1 case of hemorrhage(grade III) who was controlled with embolization of renal artery. A total of 21 complications were observed in the FURS group,including 5 cases of renal colic(grade I), 12 cases of fever over 38.5 ℃(grade II), 2 cases of Urine leakage(grade II), and 2 case of pyemia (grade II) who was controlled with sensitive antibiotics.

## Discussion

In the present study, the MPCNL group demonstrated considerably reduced operation time, extended hospitalization time, and a higher rate of stone clearance compared to the FURS group.There were no substantial disparities observed in the complication rate between the two groups.

The optimal treatment of upper ureteral calculi has been a subject of ongoing debate, but the ultimate objective is to achieve a situation where patients are completely free of stones. Aside from the overall clinical characteristics of patients, the stone-free rate and complications are also influenced by the surgeon’s expertise and the treatment options available [11]. Minimally invasive therapies like SWL, URS, PCNL, and laparoscopic ureterolithotomy are gradually replacing traditional open surgery as a result of advancements in surgical techniques and equipment.

SWL is the recommended approach for treating ureteric stones [12] due to its noninvasive nature, outpatient setting, and lack of requirement for anesthesia or surgical intervention [5]. However, when it comes to stones that are trapped and causing inflammation or have polyps, therapy with SWL may worsen swelling of the nearby mucosal tissue and is challenging to achieve the desired effectiveness. Moreover, there is a potential danger of causing harm to the renal parenchyma [13]. Moreover, when the diameter of the stone in the upper segment of the ureter exceeds 10 mm, the effectiveness of SWL in removing the stone decreases significantly [14]. Therefore, alternative minimally invasive techniques such as PCNL and URS are replacing SWL in certain individuals.

PCNL, a minimally invasive therapeutic procedure that avoids the human cavity and causes minimal harm to tissues and organs, is commonly employed for treating ureter and kidney stones [15]. PCNL has significantly elevated stone-free rates while simultaneously decreasing surgical morbidity in comparison to open stone surgery [16]. Nevertheless, patients undergoing PCNL treatment experienced noteworthy sequelae, including urine incontinence, uncontrolled bleeding, and sepsis, as reported in studies [15, 17, 18]. MPCNL, which stands for “Minimized Percutaneous Nephrolithotomy,” was created to make using larger nephroscopes and their access tubes less problematic. This procedure involves using a specialized optical puncture system called the “all-seeing needle” to perform percutaneous renal access and stone fragmentation in a single step with direct visualization. The procedure is carried out through a 4.8-foot sheath. The current investigation identified several problems associated with MPCNL, including renal pelvic damage, renal colic fever, and bleeding. While the complication rate did not show a significant difference, the MPCNL group exhibited a substantially greater incidence of bleeding, consistent with prior research [19, 20]. MPCNL remains highly invasive, even when using a smaller tract.

Currently, there is a growing preference for using holmium-YAG laser lithotripsy with FURS (flexible ureteroscopy) over semi-rigid URS (ureteroscopy) with lithotripsy for the endoscopic treatment of ureteral stones [5]. FURS, or Flexible Ureteroscopy, is a surgical procedure that utilizes the natural cavities of the human body. This method has the benefit of increased safety when treating stones near the ureter, and patients experience a swift recovery following the surgery [21]. The duration of hospitalization in the FURS group was significantly shorter compared to the MPCNL group in our study. Nevertheless, our investigation revealed that the FURS group had a longer duration of operation compared to the MPCNL group. The intricate manipulation of FURS, the need for more precise fragmentation, the assistance of a colleague, and appropriate watering may result in a longer operation time compared to MPCNL [19]. In the present era of endourology, the complication rate and morbidity associated with ureteroscopy have been considerably diminished [22]. The prevailing problems observed in URS procedures were fever and hematuria, with the majority of complications falling within categories I and II [5, 23, 24]. In this investigation, the predominant problem seen was fever, which aligned with findings reported in previous literature. While URS showed a safety advantage compared to PCNL, it had a lesser stone-free rate advantage than PCNL [21]. The stone-free rate of flexible ureteroscopy (FURS) in the current study was 71.5%, which was significantly lower compared to that of percutaneous nephrolithotomy (MPCNL) at 90.8%.

The guidelines suggest that percutaneous nephrostomy (PCN) procedures should not be performed after MPCNL in uncomplicated cases. The decision to place a PCN depends on various factors, including the presence of residual stones, the likelihood of a second-look procedure, intraoperative bleeding, perforation, ureteral obstruction, potential bacterial infection due to infected stones, the presence of a solitary kidney, and bleeding diathesis [22]. As a customary procedure that has been employed for many years at our institution, the PCN was conducted upon completion of the surgeries and was subsequently removed three days post-surgery for the patients included in this study. It is typically advised for patients to schedule their first follow-up appointment at the hospital within 2–4 weeks after surgery. During this visit, the stent is removed via cystoscopy. The duration of hospitalization for both cohorts in our investigation exceeded that reported in prior studies [5, 21, 23, 24]. The primary factor was that the patient’s preoperative examinations were carried out subsequent to their admission to the hospital. Per our municipality’s healthcare policy, reimbursement for the examination expense is only possible following hospitalization. Due to the ample availability of beds in our hospital, we did not enforce stringent control over the duration of the hospital stay.

Prior to surgery, it is imperative to evaluate the presence of stone impaction, urinary tract infections, and ureteral polyps. Prior to initiating any treatment, it is imperative to do a urine culture or urinary microscopy in order to ascertain the existence of urinary tract infections. Performing intravenous urography was essential in order to ascertain the existence of stone impaction. The preoperative identification of ureteral stones with polyps is a challenging issue. Based on our expertise, it is advisable to consider the presence of ureteral stones with polyps in the following scenarios: (1) ureteral stones tend to remain in a fixed position for an extended period, particularly when they are small, there is inadequate elimination of drugs, and there is significant hydronephrosis; (2) retrograde urography reveals the presence of filling defects or bar shadows in the ureter lumen near or below the stone; (3) urinary computed tomography shows thickening of the ureteric wall surrounding the stone and the presence of indistinct tissue shadows below the stone in patients with hydronephrosis.

Both MPCNL and FURS are viable and secure surgical alternatives for patients with solitary upper ureteral calculi measuring 1 to 2 cm, and the merits and drawbacks of these two surgical treatments have been previously deliberated. Based on our assessment, we suggest that FURS is a more preferable option for treating uncomplicated upper ureteral calculi due to its benefits of shorter hospitalization duration and faster recovery. MPCNL is the preferred option for complex upper ureteral calculi, including those with impacted stones, big stones, severe hydronephrosis or urinary tract infections, where stone removal is anticipated to be challenging. In addition, the expertise of surgeons, the state of equipment, and the preferences of the patient should also be taken into account while making treatment selections.

This study has several limitations. First,our study is a retrospective cohort study that depends on data available from medical record reviews for identification of operation time, hospitalization time,stone-free rate, and complication rate. These limitations might affect both groups and have an influence on comparing outcomes between the two groups. Second, our medical record review was unblinded, which could have led to bias in determining complications. Third,this study was a single-center study with a relatively small number of included patients and a selection bias.

Ultimately, both MPCNL and FURS are viable and secure surgical alternatives for individuals with solitary upper ureteral calculi measuring 1 to 2 cm. FURS has shown a reduced duration of hospitalization compared to MPCNL, albeit with a considerably lower rate of successful stone removal and a longer duration of the surgical procedure. There were no substantial disparities observed in the complication rate between the two groups. FURS is the preferred method for treating uncomplicated upper ureteral calculi, whereas MPCNL is the preferred method for treating complicated upper ureteral calculi. Before making treatment options, it is important to take into account the expertise of surgeons, the state of the equipment, and the preferences of the patient.

## Data Availability

The data that support the findings of this study are available from the corresponding author upon reasonable request.

## Abbreviations

MPCNL: micropercutaneous nephrolithotomy
FURS: flexible ureteroscopy
SWL: shock wave lithotripsy
